# Effects of Extrusion Conditions and Oil Addition on the Characteristics of Cheese‐Flavored Corn Snacks and Food Bolus Formation and Properties

**DOI:** 10.1111/jtxs.70102

**Published:** 2026-07-05

**Authors:** Juliana de Carvalho Marchesin, Christian Salles, Ana Carolina Conti

**Affiliations:** ^1^ Department of Food Engineering and Technology Institute of Biosciences, Humanities and Exact Sciences (IBILCE), Campus São José do Rio Preto, São Paulo State University (UNESP) São José do Rio Preto São Paulo Brazil; ^2^ Institut Agro, CNRS, INRAE, UMR CSGA, Université Bourgogne Europe Dijon France

**Keywords:** food bolus, food oral processing, image analysis, number of particles, temporal dominance of sensations, thermoplastic extrusion

## Abstract

Texture perception is a critical sensory attribute of extruded foods, such as snacks and breakfast cereals. Texture perception during eating is generated by food oral processing, depending not only on oral physiology but also on food composition and structure and their interactions. Expanded snacks have low nutritional value mainly due to their low protein and fiber content and high lipid content and energetic value, which should be improved to ensure good consumer appreciation. Thus, a rotational central composite design was applied to study the oral processing of cheese‐flavored corn snacks. The independent variables were moisture content of corn grits, extrusion temperature, and percentage of oil added (17 assays). The dependent variables included the chemical and physical properties of the products and the formation and properties of the corn snack boluses. The dynamic sensory profile of the products was also evaluated. The increase in the moisture content of corn grits reduced the number of particles in the food boluses. The increase in extrusion temperature reduced the density and force peak of snacks and chewing cycles, whereas the role of sunflower oil addition is more complex. The sensory profile of corn snacks stood out by hardness and crispness in the beginning of mastication, and sticky from the middle to the end, as well as by salty taste; however, the temporality had a low importance to characterize them. In conclusion, the sunflower oil has little impact on the sensory profile of corn snacks, suggesting that a low content of sunflower oil may be sensorially acceptable, contributing to nutritionally improved corn snacks.

## Graphical abstract

The independent variables influenced the oral processing of corn snacks, and temporality had a low importance in characterizing them. The sunflower oil content had little impact on their sensory profile, suggesting that low sunflower oil content may be sensorially acceptable, contributing to improved nutritional value of corn snacks.

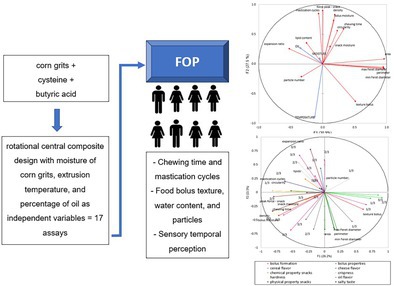

## 1 Introduction

In the human diet, food oral processing (FOP) plays a crucial role in food restructuration in the mouth, flavor release and perception, encompassing the stages of first bite, mastication, mixing with saliva, bolus formation, and swallowing (Chen 2015). This process impacts not only the temporal perception of flavor and texture of foods but also the absorption and utilization of nutrients. Constant demands exist for healthier foods, including more vegetables in the diet. These demands imply major scientific and technical advances in food reformulation. Therefore, a thorough understanding of the mechanisms involved in FOP is essential for developing food of acceptable quality to consumers (Chen 2009; Wang et al. 2024).

Mastication represents the first stage of digestion, which promotes the breakdown of foods into a bolus that can be swallowed and digested. In addition, body–food interactions in the oral phase create specific sensory experiences that underlie consumer preference and liking for food products (Chen 2015; De Lavergne et al. 2021). Therefore, investigations into the structural breakdown of foods during consumption are timely and highly relevant for understanding both eating processes and sensory perception, ultimately contributing to the development of high‐quality and palatable food textures (Chen 2015).

Among the products in which texture is a critical sensory attribute and of particular interest for investigation through FOP, extruded foods, such as snacks, breakfast cereals, and texturized vegetable proteins, represent a market segment experiencing continuous growth. Thermoplastic extrusion is a processing technology that combines the mixing of raw materials with cooking under high pressure, temperature, and shear within a barrel. The resulting mass is forced through a die, where superheated water evaporates due to the pressure drop, resulting in an expanded product with low water activity and air vacuoles in its structure. This generates a highly relevant characteristic texture for these products, constituting a critical sensory attribute responsible for consumer acceptance or rejection (Leonard et al. 2020).

In extruded products, structural characteristics, such as porosity, cell wall thickness, density, and expansion, directly influence fracture mechanics and the generation of acoustic events during biting, which are strongly associated with sensory perceptions, such as crispness, hardness, crunchiness, and mouthfeel. Recent studies have further shown that instrumental texture measurements, including force–time fracture patterns, acoustic responses, and oral tribology, can be integrated with sensory perception to better understand the mechanisms governing oral processing behavior (Aussanasuwannakul and Kantrong 2026). However, many extruded products, especially corn snacks, are frequently associated with low nutritional quality because their overall nutritional profile is typically characterized by low protein and dietary fiber contents and high lipid content and energetic value. Nevertheless, lipids influence texture, lubrication behavior, flavor release, and oral processing characteristics, making market trends contradictory because consumers are looking for a practical and, above all, a healthy diet (Menis‐Henrique et al. 2019; Ziena and Ziena 2022).

Few studies have investigated the oral processing of extruded products, both in vivo and in vitro, such as extruded flakes (Alam et al. 2017), pea flour‐based snacks (Kristiawan et al. 2021), and meat‐flavored texturized soybean protein (Conti et al. 2025). A recent study investigated the ability of an in vitro device to simulate oral functions and release taste compounds from cheese‐flavored corn snacks (Monod et al. 2025). All these studies revealed a high degree of complexity that requires deeper investigation. For instance, only the presence or absence of oil or specific extrusion conditions was tested, whereas no studies using rotational central composite designs were found to understand the effects of some independent variables on the FOP. Considering the contribution of this type of design, the need to go deeper in FOP investigations, the importance of extruded products in the market, and our expertise in this kind of product, we applied a rotational central composite design to study the oral processing of cheese‐flavored corn snacks.

## 2 Materials and Methods

### 2.1 Materials

We used corn grits (Zanin, Ibiporã, PR, Brazil), salt (Cisne, Cabo Frio, RJ, Brazil), sunflower oil (Liza, Mairinque, SP, Brazil), and monosodium glutamate (Aji‐no‐moto, Limeira, SP, Brazil). Cheese aroma precursors included L‐cysteine HCl anhydrous (purity > 98.6%, Lepuge Insumos Farmacêuticos Ltda., São Bernardo do Campo, SP, Brazil) and food‐grade butyric acid (code W222119, purity > 99%, Sigma‐Aldrich, Milwaukee, WI, USA). Artificial saliva was prepared using the following reagents from Sigma‐Aldrich (Saint Louis, MO, USA): sodium bicarbonate (NaHCO₃), dibasic potassium phosphate trihydrate (K_2_HPO_4_·3H_2_O), sodium chloride (NaCl), potassium chloride (KCl), calcium chloride dihydrate (CaCl_2_·2H_2_O), sodium azide (NaN_3_), mucin, and α‐amylase (Menis‐Henrique et al. 2019).

### 2.2 Extrusion of Corn Grits and Corn Snacks Preparation

Corn grits were extruded using a single‐screw extruder (model RXPQ Labor 24, Indústria de Máquinas INBRAMAQ Ltda., São Paulo, Brazil), equipped with five independent heating zones, under the following conditions: helicoidally grooved barrel; screw with a large step of one exit, compression ratio of 3.3:1 and length‐to‐diameter (L/D) ratio of 15.5:1; pre‐die with holes of 3.03 mm; die with a diameter of 2.99 mm (round hole); feed rate of 265 g/min; screw speed of 192 rpm; temperatures in zones from 1 to 3: off (40°C), 70°C and 90°C.

The moisture content of the corn grits (dry basis), the temperature of zone 5 of the barrel, and the quantity of sunflower oil sprinkled on the extrudate varied according to a rotational central composite design for three independent variables with five levels each: moisture content (10, 12, 15, 18, and 20 g/100 g), zone 5 temperature (90°C, 102°C, 120°C, 138°C, and 150°C), and sunflower oil (0, 5, 12, 19, and 24 g/100 g of extrudate). Seventeen assays were performed: eight assays of factorial points, six assays of axial points, and three assays of the central point. The dependent variables were the chemical characteristics and physical properties of the corn snacks and the food boluses. This design was based on previous studies by the research group (Flôres et al. 2024; Menis‐Henrique et al. 2020). The sequence of the assays was established from the lowest to the highest temperature, and the tests were randomized for the same temperature.

First, the moisture content of corn grits was determined in a hot‐air oven at 105°C (AOAC 1997) and subsequently adjusted according to the central composite rotational design. After verifying the adjustments of all moisture levels in the oven at 105°C (AOAC 1997), corn grits were added with butyric acid (0.4/100 g) and cysteine (0.2/100 g). The addition of butyric acid was performed by volume, based on its density, while cysteine (powder) was added by weight. The mixtures were homogenized, stored in polyethylene bags, and kept under refrigeration. The bags were removed and held at 22°C for 2 h prior to extrusion.

After extrusion, the extrudates were cut into 5‐cm pieces with a mold and added with sunflower oil in the amounts defined by the experimental design, salt (1.4/100 g of extrudate), and monosodium glutamate (0.6/100 g of extrudate) according to Menis‐Henrique et al. (2020). Snacks were packaged in metalized polyester film bags without a modified atmosphere and stored at 22°C until further analysis.

### 2.3 Analysis of the Chemical Characteristics and Physical Properties of the Corn Snacks

The moisture (triplicate) and lipid (duplicate) contents of the snacks were determined by drying at 105°C (AOAC 1997) and Bligh and Dyer (1959), respectively.

Regarding the physical properties, corn snacks were evaluated (10 replicates) for the following:

– Expansion ratio, determined as the ratio between the diameter of the extrudate and the diameter of the extruder die using an IP54 digital caliper (Digimess, Brazil).
– Density, calculated from the extrudate diameter (*D*, cm) and length (*L*, cm) measured with the same caliper, and weight (*W*, g) using the following equation: *ρ* = 4*W*/*πD* ^2^ *L*.
– Instrumental texture was measured using a TA.XT/Plus/50 texture analyzer (Stable Micro Systems, Surrey, UK) equipped with an AED (Stable Micro Systems, Surrey, UK) to capture the acoustic emission of snack fracture (Dias‐Faceto et al. 2020). A three‐point bending probe (HDP/3PB) was used, and 1.1 mL of artificial saliva, prepared according to Menis‐Henrique et al. (2019), was applied to the top of the sample using a 3 mL disposable syringe. A waiting time of 7 s was set before the start of the test, with pre‐test and test speeds of 2 and 1 mm/s, respectively. The force peak obtained was considered the cutting force of the snack, and the sound pressure level was calculated as the mean of the 10 highest acoustic peaks (Guazi and Conti 2023).

### 2.4 Oral Processing of the Corn Snacks

This study was approved by the Research Ethics Committee of IBILCE/UNESP (opinion number 6.485.081), and all panelists provided informed consent. The analysis was conducted in individual booths under white light, at 22°C. Eight healthy panelists (six women and two men, aged 20–47 years) were recruited to chew and evaluate the samples, all of whom were regular consumers of the product, with no food allergies, intolerance, dental (missing teeth, use of prosthesis or orthodontic device, or piercings), mastication problems, or salivary disorders. Samples were presented in a balanced and monadic manner and coded with three random digits. All samples were analyzed in three repetitions, and because of this, each panelist participated in 10 sessions, following the procedure used by Conti et al. (2025).

#### 2.4.1 Session 1: Oral Characterization of the Panelists

The first session was conducted to characterize the panelists by measuring salivary flow and mastication performance. The salivary flow was stimulated by chewing a 5 × 5 cm piece of Parafilm (Parafilm M, Bemis Company Inc., Chicago, IL, USA) for 5 min, and the saliva was collected in disposable aluminum containers (Gavião et al. 2004) in three repetitions. Salivary flow (mL/min) was calculated as the difference in the aluminum container mass with and without saliva, considering that human saliva has a density of 1.0 g/mL (Richardson and Feldman, 1986, cited by Gavião et al. 2004). The stimulated salivary flow ranged from 0.7 to 3.2 mL/min, all within normal levels (Table S1).

Subsequently, the panelists chewed a piece of raw carrot (2 cm in diameter and 1 cm in height) 10 times at a normal rhythm (Woda et al. 2010) in three repetitions to assess mastication performance. The panelists were instructed to spit the carrot bolus into a 250 mL disposable plastic container, rinse their mouth three times with a small amount of water to remove particles adhering to the teeth, and spit the carrot bolus into the same container again. The carrot boluses were image‐analyzed. For this, water was added to each bolus to a final volume of 150 mL, homogenized, and gently transferred onto a glass plate. Particle images were obtained using an HP Deskjet Ink Advantage 1516 scanner in black and white scanning at 1200 dpi and subsequently analyzed using ImageJ software (National Institutes of Health, Bethesda, MD, USA) for particle count and size distribution. Particles with an area smaller than 0.16 mm^2^ were excluded, and the area and minimum Feret diameter were determined for each remaining particle and used to calculate d50 as an indicator of mastication performance. The particle size (*d50*) of the carrot boluses of the panelists ranged from 6.4 to 12.8 mm (Table S1), which is not very different from those reported in the literature (Woda et al. 2010).

#### 2.4.2 Sessions 2–4: Evaluation of Food Bolus Texture and Water Content

In these sessions, the panelist chewed the samples at a normal rhythm until just before swallowing, and the food bolus was spit into a disposable aluminum container. The panelist counted the number of chewing cycles, and the researcher used a stopwatch to measure the chewing time.

Immediately after each session, the collected food bolus was evaluated for texture using a TA.XT/Plus/50 texture analyzer (Stable Micro Systems, UK) and Texture Exponent 32 software (Stable Micro Systems, Godalming, UK) equipped with a 10‐mm‐diameter aluminum cylindrical probe under the following test conditions: compression of 2 mm, pretest speed of 2 mm/s, test speed of 1 mm/s, and posttest speed of 10 mm/s. The obtained peak force was considered the force necessary to compress the food bolus. A mold was used to retain the food bolus during the analysis. Thus, the probe and the mold were rinsed with 1 mL of water after the analysis.

Subsequently, the aluminum container was placed in an oven at 103°C for 24 h to determine the moisture content of the food bolus by evaporation (Drago et al. 2011). The mass of the water previously used for rinsing the probe and mold during the texture analysis was disregarded for this measurement.

#### 2.4.3 Sessions 5–10: Evaluation of Temporal Perception and Bolus Particles

In these sessions, the panelist chewed the samples at a normal rhythm until just before swallowing and was instructed to:

– Evaluate the samples using the Temporal Dominance of Sensations (TDS) technique (Pineau et al. 2009) and SensoMaker software (Nunes and Pinheiro 2012). The texture and flavor attributes were selected based on the work of Flôres et al. (2024), who evaluated similar corn snacks produced from corn grits extruded under different conditions but with the same aroma precursors. In this study, nine attributes were raised for the Rate‐All‐That‐Apply test (hardness, crispness, sticky, salty taste, umami taste, cheese flavor, oil flavor, and cereal flavor); however, SensoMaker allows up to eight attributes. Thus, the burnt flavor was discharged because it did not represent the samples, and hardness, crispness, sticky, salty taste, umami taste, cheese flavor, oil flavor, and cereal flavor were used. The panelists were familiarized with the TDS evaluation procedures and how to use the SensoMaker. They were also familiarized with the attributes used because they were regular consumers of the product, and TDS can be applied with non‐trained panelists (Pineau and Schlich 2015). Considering the chewing times for snacks from 13.8 to 17.3 s (see Section 3.2), the analysis was run for 30 s.
– Spit the food bolus into a 250 mL disposable plastic container, rinse the mouth once with a small amount of water to remove sample residues adhered to the teeth, and spit again into the same container.

Immediately after each session, 1.5 mL of 20% sodium dodecyl sulfate (SDS) was added to the collected food bolus (except the assay with no sunflower oil) because of its surfactant properties, which linked to the oil dispersed in the water and improved the image quality, avoiding the formation of a halo around the particles that could disturb the image analyses (Conti et al. 2025). Then, water was added to a final volume of 150 mL, homogenized, and gently transferred onto a glass plate. Subsequently, the food boluses were subjected to image analysis, as described for carrots in Session 1. All these steps were performed shortly after each bolus to reduce the possibility of SDS penetration into the particles' interior, altering their structure. The number of particles in each food bolus was determined, and the following parameters were analyzed for each particle: area, perimeter, minimum and maximum Feret diameters, and circularity (Wirth 2004; Sympatec GmbH 2026). Data are expressed as medians.

### 2.5 Statistical Analysis

The linear and quadratic models were tested to explain the influence of the independent variables on the dependent variables. The results were then subjected to multiple regression analysis, and the model coefficients were considered significant when *p* ≤ 0.05 and a model adjustment coefficient (*R* ^2^) ≥ 70% was necessary. Regression was evaluated by ANOVA, with regression being significant when *p* ≤ 0.05 and without lack of fit when *p* > 0.05. Statistica 7.0 (StatSoft Inc., USA) and Minitab 21 (Minitab Inc., USA) were used for data analysis.

TDS curves were obtained using the SensoMaker software (Nunes and Pinheiro 2012), and attributes were considered dominant when above the significance level of 0.05. Moreover, to obtain quantitative data from this analysis, the 30 s of analysis were divided into three periods (from 0 to 10 s, from 11 to 20 s, and from 21 to 30 s), and the number of times that each attribute was indicated as dominant (represented by the number 1 by the SensoMaker) was counted.

Multivariate analyses were performed to identify correlations among variables. Principal component analysis (PCA) was performed on the chemical and physical properties of the snacks and the formation and properties of the bolus particles. The independent variables were considered supplementary variables in the PCA. Multiple factor analysis (MFA) was run for TDS results and with variables from the snacks and boluses. Pearson correlation was also performed for specific variables to enrich the discussion of the data. These analyses were performed using the XLSTAT statistical software for Microsoft Excel (Addinsoft, USA).

## 3 Results and Discussion

### 3.1 Chemical and Physical Properties of the Corn Snacks

**FIGURE 1:**
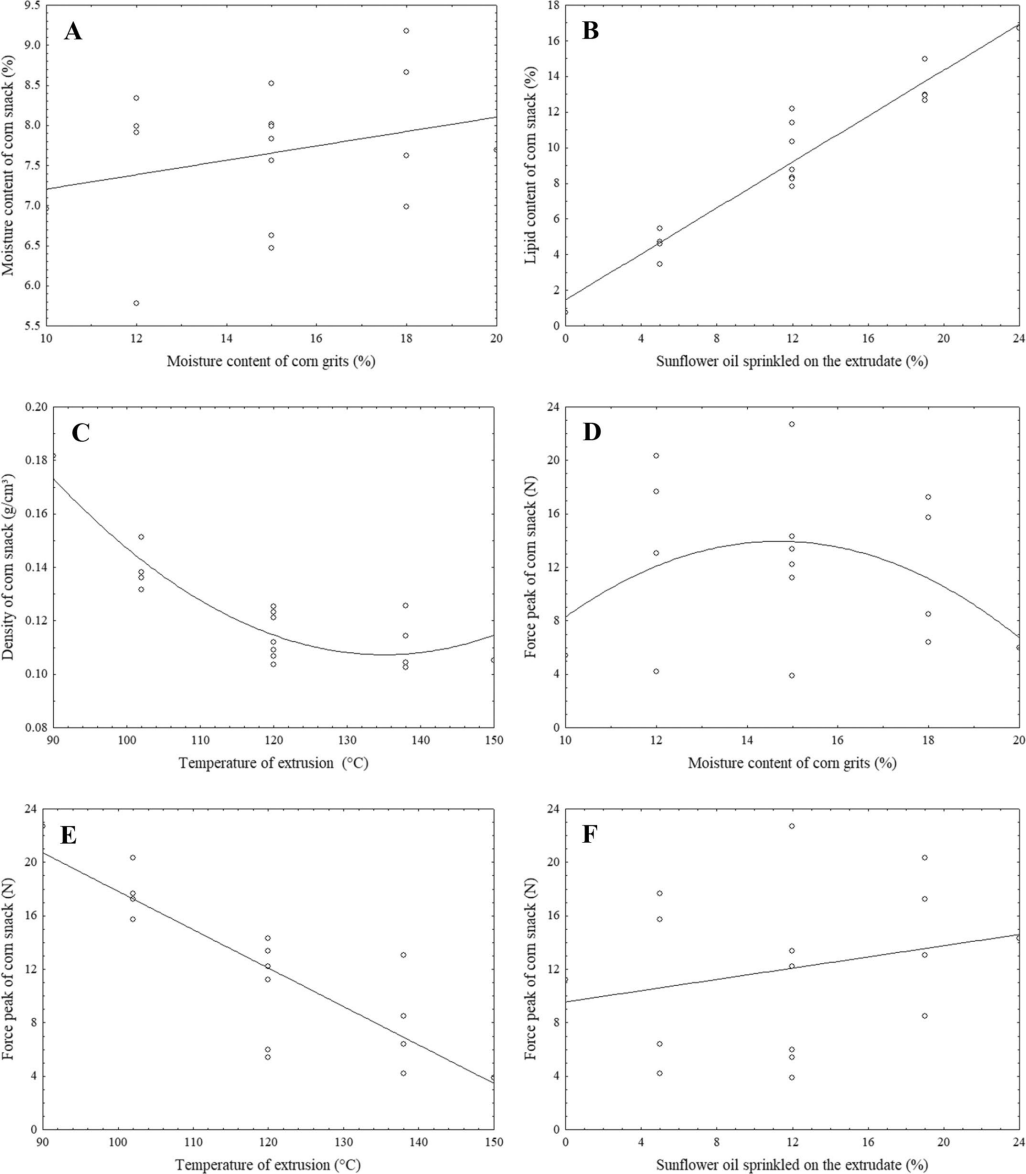
Moisture (A) and lipid (B) contents, density (C), and force peak (D–F) of corn snacks as functions of independent variables.

The moisture and lipid contents of the corn snacks ranged from 5.8% to 9.2% and from 0.7% to 16.7%, respectively (Table S2). Table 1 presents the best‐fitted models for the moisture and lipid content of the snacks. The increase in the moisture content of corn grits or the decrease in the extrusion temperature or the percentage of sunflower oil increased the moisture content of corn snacks (Figure 1A; only the graph for corn grits moisture is shown). An increase in the moisture content of corn grits may hinder water evaporation during and at the end of the extrusion process, resulting in higher moisture levels in the final product. In contrast, higher extrusion temperatures enhance water evaporation, reducing the moisture content. The effect of sunflower oil addition is less evident to interpret. This effect is attributed to the increase in the mass of the extrudate. As sunflower oil addition increases, the relative proportion of extrudate in the sample analyzed in the oven drying decreases, which may explain the lower moisture content observed in the corn snack.

**TABLE 1:** Models and goodness of fit for the dependent variables of the corn snacks.

Dependent variable	Model	Adjusted *R* ^2^ (%)	*p*	Lack of fit
Moisture content	*Y*_MC_ = 10.40 + 0.08*X*_1_–0.02*X*_2_ − 0.09*X*_3_	74.8	0.000	0.262
Lipid content	*Y*_LC_ = 2.73 + 0.64X_3_	90.1	0.000	0.760
Density	*Y*_D_ = 0.67−0.01*X*_2_ + 0.00 *X*_2_^2^	77.3	0.001	0.471
Force peak	*Y*_FP_ = 27.3 − 0.79 *X*_2_ + 0.01 *X*_3_ − 0.23*X*_1_^2^	85.0	0.000	0.071

Abbreviations: *X*_1_, moisture of corn grits; *X*_2_, temperature of zone 5 of the barrel; *X*_3_, percentage of sunflower oil sprayed on the snacks.

As expected, the lipid content of the snacks increased with an increase in the amount of sunflower oil (Figure 1B). Nevertheless, not all the sunflower oil added to the snacks was incorporated because the real lipid percentages were lower in almost all assays (Table S2). Menis‐Henrique et al. (2020) reported similar results, which can be explained by: (1) oil transfer from the snack to the container used during oil spraying; (2) oil loss due to friction among snacks during oil application and homogenization; (3) oil transfer to the packaging during storage; and (4) interference of the extrusion process on snack structure, including variations in expansion ratio, vacuole size, and snack porosity. Even though the Pearson correlation showed a strong positive correlation between the amount of sunflower oil and lipid content (*r* = 0.96; *p* < 0.0001), indicating a constant proportionality between them.

The expansion ratio and density of corn snacks ranged from 3.5 to 4.7 and 0.10 to 0.18 g/cm^3^ (Table S2). The snacks exhibiting one of the highest expansion ratios were produced using the optimal moisture content of the corn grits for extrusion (15 g/100 g) combined with an intermediate extrusion temperature (120°C). Similar findings were reported in experiments conducted within the research group (Flôres et al. 2024; Menis‐Henrique et al. 2020). The force peak ranged from 3.9 to 22.7 N, and the sound pressure level ranged from 61.3 to 95.0 dB (Table S2). These values are similar to those reported by Flôres et al. (2024). For these variables, Table 1 presents only the significant models, without lack of fit, and with an *R* ^2^ greater than 70%. The increase in the extrusion temperature reduced the product density (Figure 1C), with a slight increase observed from 138°C (+1 level of temperature in the experimental design) due to the quadratic effect of temperature. High extrusion temperatures promote greater evaporation of superheated water during processing and at the die exit, facilitating the formation of air bubbles and reducing product density (Fellows 2000). Similar results were reported by Saeleaw et al. (2012), Menis‐Henrique et al. (2013), and Menis‐Henrique et al. (2020).

The moisture content of the corn grits had a quadratic effect on the force peak of the snacks (Figure 1D), with the central point showing the highest force. In addition, decreasing the temperature or increasing the sunflower oil content increased the force peak (Figure 1E,F, respectively). A decrease in the extrusion temperature increases the melt viscosity, hindering the growth of air bubbles and resulting in thicker cell walls in the extrudates (Yuliani et al. 2006), thereby increasing the cutting force. The effect of sunflower oil may be attributed to its adhesion to the snack surface, causing slight moistening and increasing the force required to fracture it. A similar effect was found when artificial saliva was used during the instrumental analysis of texture (Guazi and Conti 2023). This was because the extruded sample was moistened by the saliva and did not break easily; rather, it was “crushed” by the probe, thus increasing the force.

### 3.2 Formation and Properties of the Corn Snack Boluses

**FIGURE 2:**
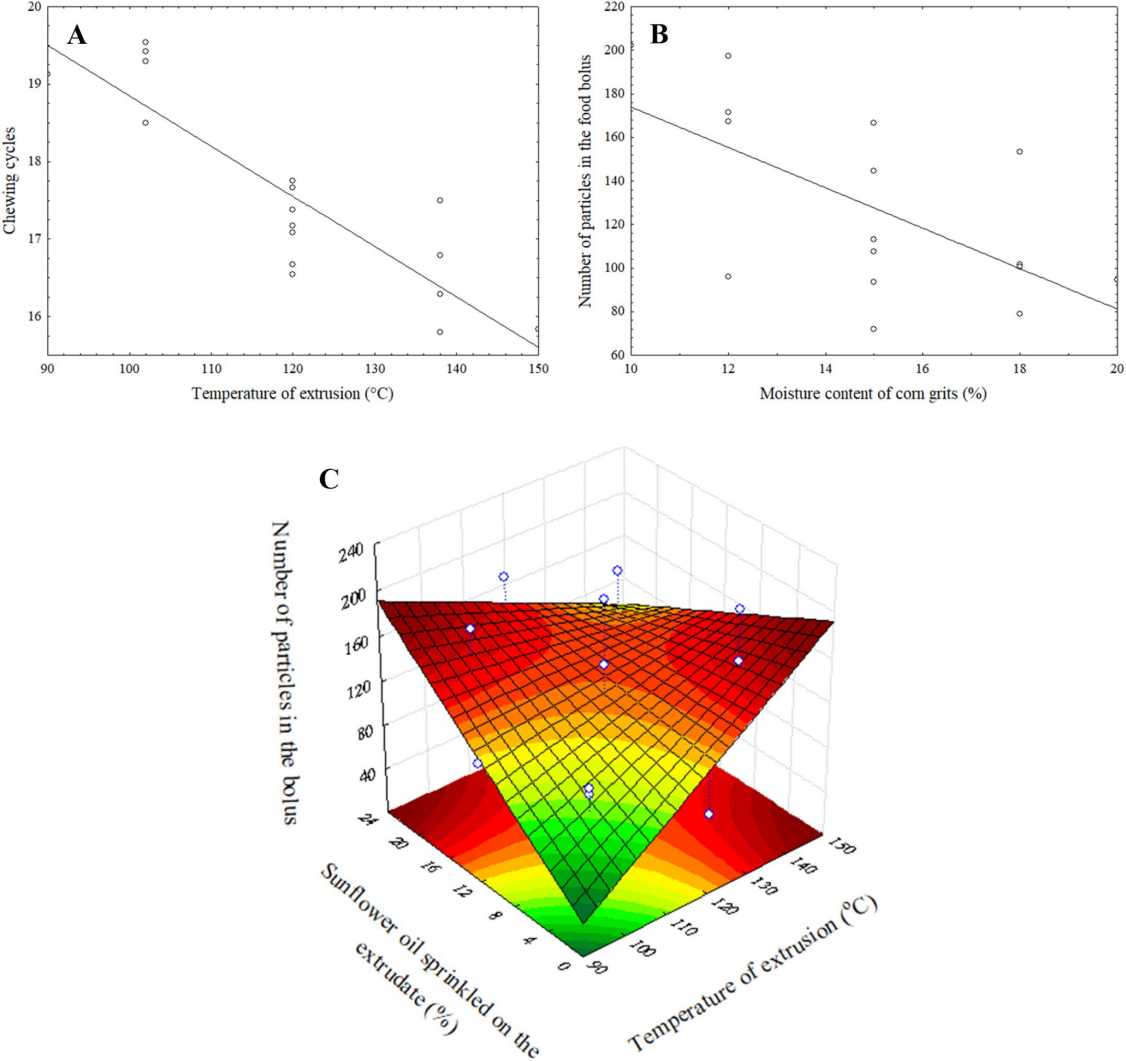
Chewing cycles (A) of corn snacks and the number of particles in food boluses (B and C) as functions of independent variables.

The chewing time ranged from 13.8 to 17.3 s, the number of chewing cycles from 15.8 to 19.5, the bolus moisture content from 24.8% to 31.3%, and the force peak for bolus compression from 0.17 to 0.32 N (Table S3). The only significant model without lack of fit and with an *R* ^2^ greater than 70% was obtained for the number of chewing cycles (Table 2). An increase in the extrusion temperature reduced the number of chewing cycles required for swallowing the corn snack bolus (Figure 2A). This effect may be related to the lower product density, since increasing the temperature also reduced the snack density (Figure 1C). Less dense expanded snacks have a structure with thinner internal walls (Meng et al. 2010; Menis‐Henrique et al. 2020), which may facilitate their breakdown during mastication, thus requiring fewer chewing cycles. Indeed, this positive correlation, through PCA and MFA, will be further discussed.

**TABLE 2:** Models and goodness of fit for the dependent variables of the bolus formation and particles.

Dependent variable	Model	Adjusted *R* ^2^ (%)	*p*	Lack of fit
Chewing cycles	*Y*_MC_ = 24.15 − 0.07*X*_2_	75.4	0.000	0.537
Number of particles	*Y*_NP_ = −483 + 18.1*X*_1_ − 0.18*X*_2_*X_3_	53.3	0.025	0.534

Abbreviations: *X*_1_, moisture of corn grits; *X*_2_, temperature of zone 5 of the barrel; *X*_3_, percentage of sunflower oil sprayed onto the snacks.

The boluses obtained from the different assays contained between 72 and 202 particles, with an area ranging from 0.5 to 22.9 mm^2^, perimeter from 11.2 to 36.1 mm, a minimum Feret diameter ranging from 0.9 to 2.6 mm, a maximum Feret diameter ranging from 1.6 to 3.8 mm, and a circularity ranging from 0.05 to 0.06 (Table S4). A few particles were found, which is characteristic of the sample, which is rich in starch. The lubrication of the sample with saliva during mastication and the digestion of starch by the α‐amylase (Goff et al. 2018) makes the product sticky and agglomerates the food bolus.

In contrast to what was expected, no model for any particle variable met all statistical criteria. Nevertheless, the model for the number of particles was significant and did not lack fit (Table 2). Although the model obtained for the number of bolus particles showed a moderate adjusted *R* ^2^ value (53.3%), it remained statistically significant and without lack of fit, indicating that the selected independent variables contributed meaningfully to explain part of the variability associated with particle formation during oral processing. The relatively low model fit quality may be attributed to the intrinsic complexity and multifactorial nature of oral processing phenomena, which are influenced not only by food composition and structure but also by dynamic physiological factors, such as saliva incorporation, mastication strategy, tongue movements, individual oral behavior, and particle agglomeration during bolus formation. Furthermore, additional interaction and quadratic terms were evaluated during the statistical modeling step; however, their inclusion did not substantially improve the model fitting while maintaining statistical significance and without lack of fit. Therefore, the simplest statistically valid model was retained. Despite its moderate fit quality, this result remains relevant considering the exploratory and innovative nature of combining response surface methodology with oral processing variables in vivo, an approach that is still scarcely explored in the literature. The number of particles in the boluses decreased as the moisture content of corn grits increased (Figure 2B). The increase in grit moisture also increased the moisture content of the snacks (Figure 1A), and products with higher water and fat contents require less time in the mouth to allow sufficient saliva secretion to moisten and form a cohesive bolus before being swallowed (Gavião et al. 2004). The higher moisture content of the snack may have led to lower saliva incorporation, reducing the product's residence time in the mouth and its breakdown/fragmentation, resulting in a smaller number of particles.

Moreover, an interaction was observed between the extrusion temperature and the addition of sunflower oil. The number of particles was higher either at high extrusion temperature combined with low sunflower oil or at low temperature combined with high sunflower oil, whereas the number of particles was lower when both variables were high or low (Figure 2C). Nevertheless, such interaction is not so evident to interpret and leads us to consider the physical structure of the snack and its ability to absorb oil for understanding some results. When a high extrusion temperature is applied, the snack is more fragile due to the thinner walls, as explained for density and force peak (Figure 1C,E, respectively). In this condition, lower and higher percentages of sunflower oil sprinkled onto the snack result in lower and higher absorption of the sunflower oil by the snack structure, respectively (Figure 1B). When masticated, snacks with higher oil content slip in the mouth, reducing the effectiveness of mastication, destructuring less food, and producing fewer particles. The opposite is expected when a snack with a lower oil content is masticated, producing more particles.

Nevertheless, when a low temperature is used, the cooking of dough is not so effective, reducing the water evaporation and resulting in a product with thicker walls and larger vacuoles (Meng et al. 2010; Menis‐Henrique et al. 2020), which explains the harder structure (Figure 1E). Thus, the lower sunflower oil percentage sprinkled onto the snack is not sufficient to lubricate it, which would enhance the mastication friction and increase the number of particles. Under the same understanding, a lower number of particles would be found when a higher sunflower oil percentage is used. Nevertheless, we found contrary results. Other interfering factors, such as salivary flux, alpha‐amylase action, and even the fact that the sunflower oil was sprinkled on the snack's surface and not homogeneous in the whole product, may have influenced this. However, as we did not evaluate this, it should be investigated further.

**FIGURE 3:**
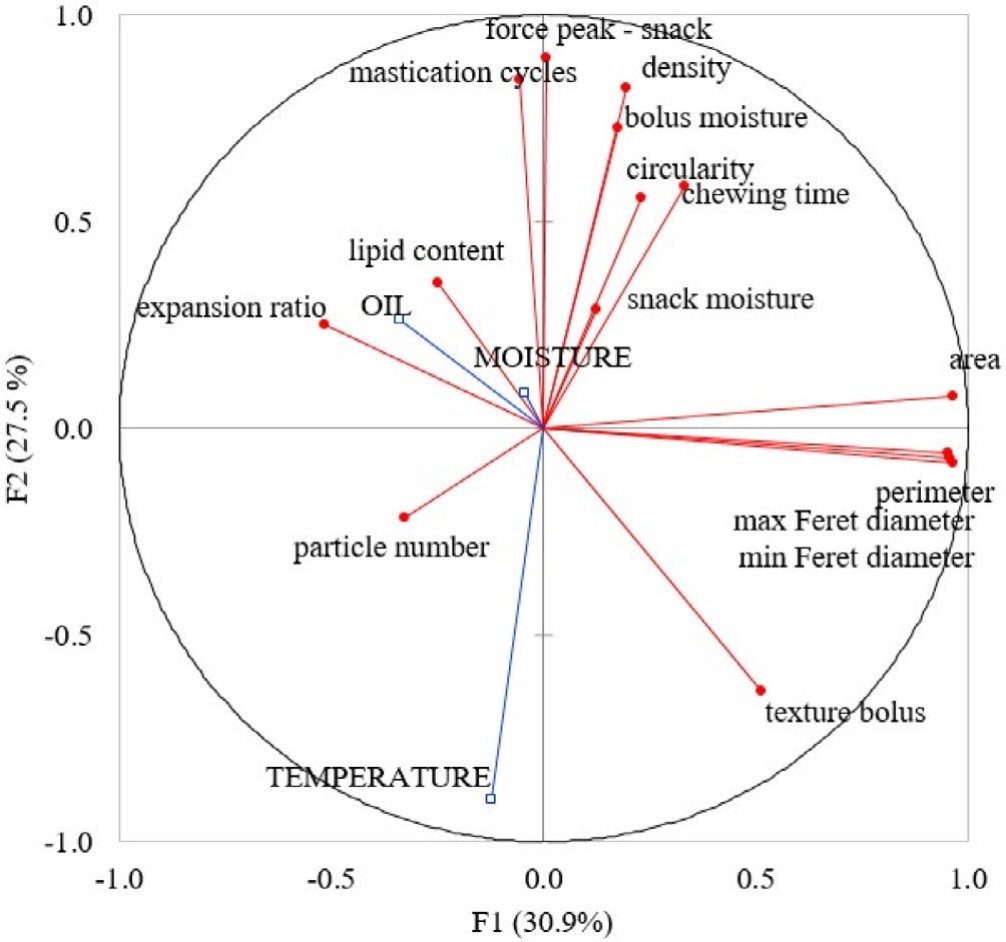
Principal component analysis of the chemical and physical properties of corn snacks and formation and properties of the food boluses. Supplementary variables: Moisture (corn grits), Temperature (zone 5 of the barrel), and Oil (sunflower oil sprayed onto the snacks).

Expansion ratio, density, and instrumental texture are adequate indicators of dough texturization during extrusion and subsequent structural characterization of corn snacks. High expansion ratio and low density are optimal characteristics for corn snacks because the expansion ratio refers to transversal expansion, whereas the density represents expansion in all directions. This results in a more brittle structure or less mechanical resistance, which are desirable characteristics for expanded snacks (Yuliani et al. 2009; Milk et al. 2021). Although explaining 58.4% of the total variation, the PCA (Figure 3) shows that temperature, as a supplementary variable, had a negative effect on the peak force and density of the snacks, as well as on the number of chewing cycles and the moisture content of the food bolus, corroborating the results presented in Figures 1C,E and 2A. In fact, denser snacks require greater force to be broken, leading to an increase in the number of chews and promoting greater moistening of the food bolus with saliva, thereby increasing their moisture content.

### 3.3 Sensory Profile of the Corn Snacks

**FIGURE 4:**
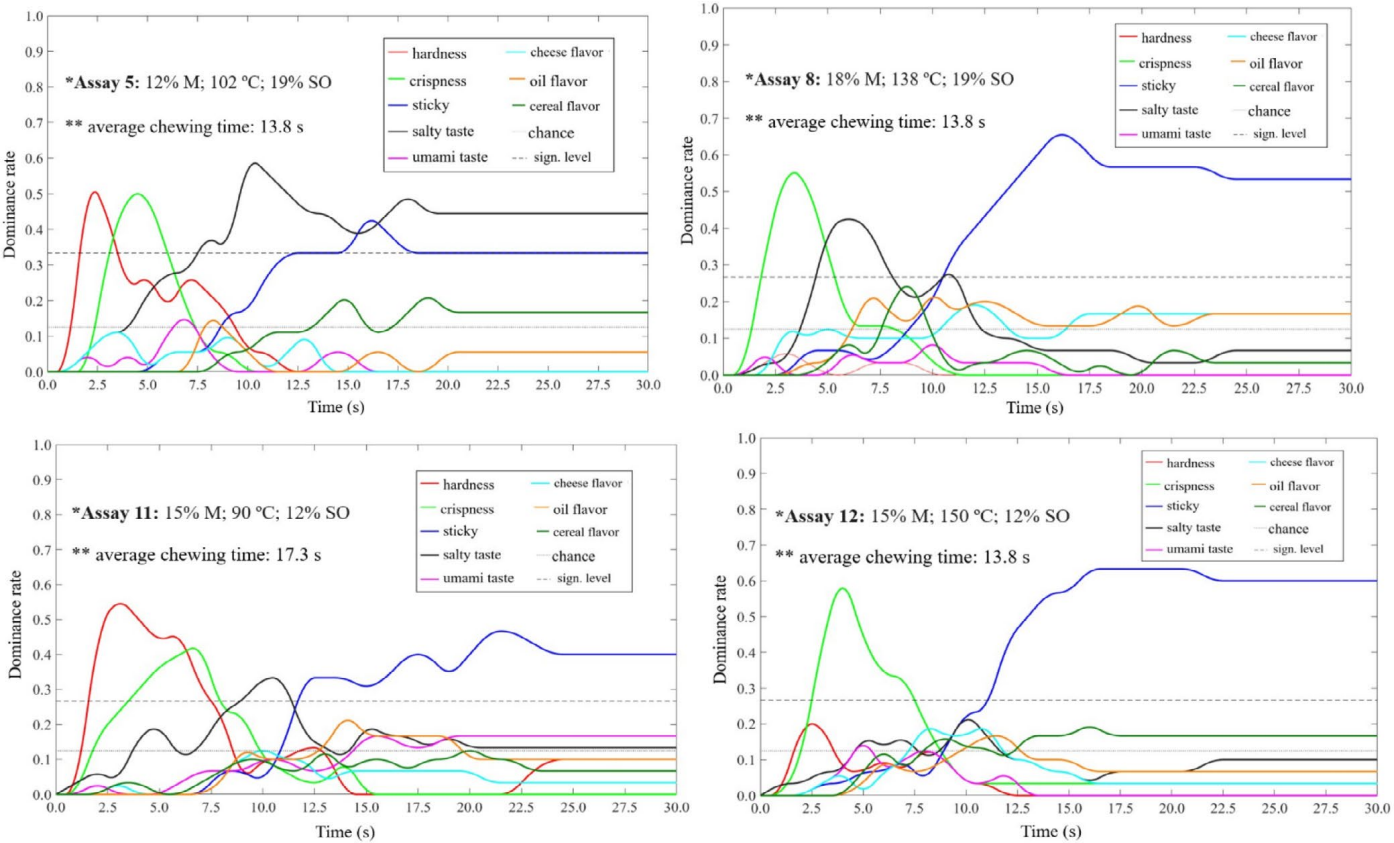
Dominance curves of the sensory attributes of the corn snacks.

Figure 4 presents the TDS curves for some samples: assays 5 (12% moisture, extrusion at 102°C and 19% sunflower oil), 8 (18% moisture, extrusion at 138°C and 19% sunflower oil), and 12 (15% moisture, extrusion at 150°C and 12% sunflower oil), which exhibited the shortest chewing times (13.8 s), and assay 11 (15% moisture, extrusion at 90°C and 12% sunflower oil), which showed the longest one (17.3 s). The other samples exhibited similar behavior, even when obtained under different extrusion conditions and sunflower oil addition. Crispness was the dominant attribute at the beginning of mastication for all samples, whereas hardness was the dominant attribute for assays 5 and 11. Both texture attributes are relevant to extruded products because they are a consequence of the adequate texturization of the dough during extrusion and evaporation of the superheated water through the extruder die, resulting in a porous and crispy product (Dias‐Faceto and Conti‐Silva 2022). The same behavior was observed in corn snacks obtained under other extrusion conditions (Menis‐Henrique et al. 2019), strengthening the importance of both attributes to this type of product.

Subsequently, the dominance was followed by a salty taste and stickiness from the middle of the chewing time until the end of the mastication (except for salty taste in assay 11). Assays 8 and 12 were characterized by the dominance of crispness at the onset of mastication, followed by the sticky attribute for approximately 10 s. Moreover, the salty taste was dominant between these attributes in assay 8. This makes sense because the product needs to be broken first so that saliva can come into greater contact with the particles, extracting the salt to be perceived. Once it is well hydrated by saliva, the product becomes sticky. Stickiness is expected in all samples because they are a starchy product, and both saliva incorporation and alpha‐amylase digestion make the product sticky (Guazi and Conti 2023).

The dominance of the other attributes was specific, and the only one that deserves a highlight is the cereal flavor to assay 13 (15% moisture, extrusion at 120°C and 0% sunflower oil). This sample had no sunflower oil addition, standing out the own flavor of cereal (or corn, in this case). Umami taste and cheese flavor were not dominant in any sample. The absence of dominance of cheese flavor was unexpected. However, butyric acid, a volatile compound with cheese‐like notes, is a lipophilic compound. Lipophilic volatile compounds need time to be sensorially perceived because they must first be released from the food matrix and migrate through the oily surface (derived from the sunflower oil sprayed onto the extrudate) into the saliva and then be released into the oral cavity (Leland 1997; Madene et al. 2006). Since the snacks required a short chewing time before swallowing (13.8–17.3 s), there may not have been sufficient time for the perception of cheese flavor. Another reason may be the high threshold of this compound, but it was not evaluated.

**FIGURE 5:**
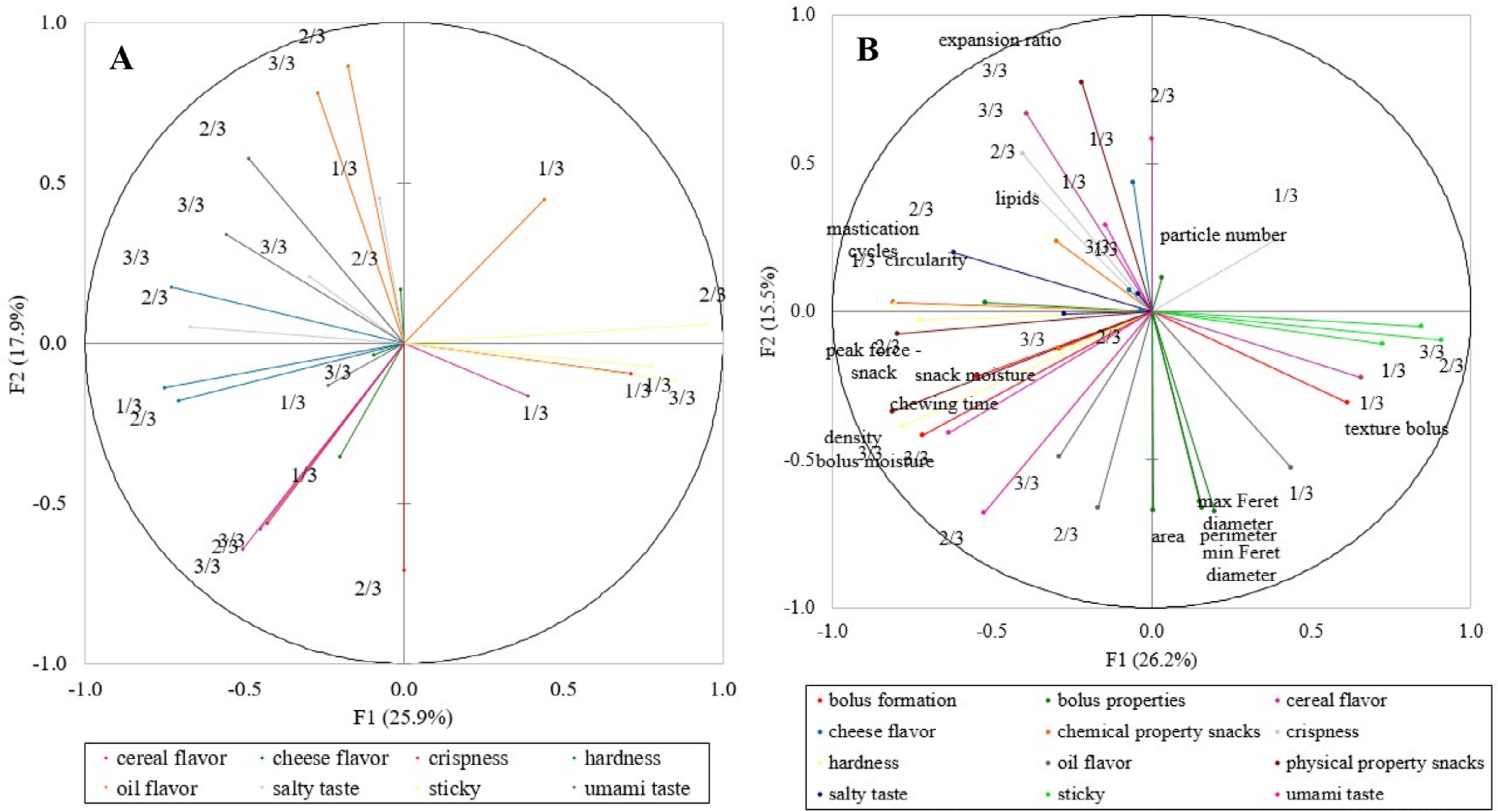
Multiple factor analysis of the sensory profile of corn snacks along the Temporal Dominance of Sensations evaluation (1/3, 2/3, and 3/3 of the total time of analysis) (A) and with chemical and physical properties of corn snacks and formation and properties of the food boluses (B).

These results may be complemented by those obtained from MFA considering the three periods during TDS analysis (from 0 to 10 s, from 11 to 20 s, and from 21 to 30 s). Although it explained only 43.8% of the total variation (Figure 5A), it is interesting to observe that variables at the three periods are near in the two‐dimensional map, which means that the temporality of sensation dominations did not describe the samples, that is, samples were described by the sensory attributes independent of the time. Simultaneously, if the variables from the snacks and the boluses are combined, the MFA (41.6% of the total variation) results are very similar (Figure 5B), with a low importance of temporality compared to the sensory attributes. These results are probably due to the short duration of the mastication of the corn snacks.

### 3.4 Limitations of the Study and Future Outlooks

Interindividual physiological variability influences oral processing, including differences in mastication patterns, saliva incorporation, tongue movements, and swallowing behavior. While stimulated salivary flow and masticatory performance were evaluated to characterize panelists, other potentially relevant physiological factors, such as salivary enzymatic activity and saliva viscosity, as well as the bolus rheology, were not evaluated and should be addressed in future studies to better explain bolus formation and perception. Although the number of panelists may seem low, the sample size was within the range commonly reported in FOP studies involving in vivo mastication and bolus collection (Fernández et al. 2024; Conti et al. 2025) due to the high experimental complexity and time demand associated with these evaluations. Moreover, we highlight that repeated measurements are important to enhance data collection, allowing adequate statistical treatment.

Another limitation is that the oil retention efficiency was indirectly inferred from the experimentally determined lipid contents rather than directly quantified. Adsorption analysis and confocal microscopy should be performed in future studies to provide more information on the properties of the snack, oral processing, and sensory perception.

Despite these limitations, this study provides relevant insights into the relationships among extrusion parameters, oil addition, snack structure, and oral processing. Future studies should include a larger sample size, more comprehensive oral physiological characterization, integration of oral processing with nutrient bioavailability and bioaccessibility measurements, and direct evaluation of oil retention and flavor release mechanisms to further improve the nutritional value of extruded snacks.

## 4 Conclusions

Although the use of the rotational central composite design to study the oral processing of cheese‐flavored corn snacks had shown few results meeting the statistical criteria, it brought interesting results. The increase in the extrusion temperature reduced the number of chewing cycles, and the increase in the moisture content of corn grits reduced the number of particles in the food boluses. The interaction between extrusion temperature and sunflower oil addition influenced the number of particles; the number of particles was higher either at high extrusion temperature combined with low sunflower oil or at low temperature combined with high sunflower oil, whereas the number of particles was lower when both variables were high or low. Although the physical structure of the snack and its ability to absorb oil can explain some of the results, others must be investigated further. The sensory profile of corn snacks stood out by the texture attributes, being hardness and crispness in the beginning of mastication, and sticky from the middle to the end, and salty taste. The cheese flavor was not dominant in any sample, and temporality had a low importance in characterizing the sensory profile of snacks. Moreover, the sensory profile of the snack without oil addition may be highlighted to present the cereal flavor as dominant, different from the others. In conclusion, the study of oral processing of food is relevant to product design and performance, especially regarding sensory aspects related to a better nutritive value. In this way, it is relevant to highlight that the sunflower oil has little impact on the corn snacks and, considering the little difference between samples, we suggest the addition of 5% of sunflower oil (the lowest amount of sunflower oil used in this study). Therefore, the study of oral processing here shows the feasibility of adding a low content of sunflower oil to snacks, contributing to nutritionally improved corn snacks.

## Author Contributions

**Christian Salles:** conceptualization, funding acquisition, supervision, writing – review and editing. **Juliana de Carvalho Marchesin:** investigation, writing – original draft, writing – review and editing. **Ana Carolina Conti:** conceptualization, funding acquisition, investigation, data curation, formal analysis, supervision, writing – original draft, writing – review and editing.

## Funding

This work was supported by São Paulo Research Foundation—FAPESP (grant 2023/11286‐4), National Council for Scientific and Technological Development—CNPq (process 303602/2022‐8), and Brazilian Federal Agency for Support and Evaluation of Graduate Education (CAPES).

## Conflicts of Interest

The authors declare no conflicts of interest.

## Supporting information


**Table S1:** Stimulated salivary flux and particle size of the carrot bolus (mean ± SD; *n* = 3).

**Table S2:** Moisture (*n* = 3) and lipid (*n* = 2) content and physical parameters of corn snacks (*n* = 10).

**Table S3:** Properties of food boluses (mean ± standard deviation; *n* = 24) of corn snacks.

**Table S4:** Particles of food boluses (mean ± standard deviation; *n* = 24) of corn snacks.

## Data Availability

The data that support the findings of this study are openly available in Repositório Institucional UNESP (after embargo of 6 months) at https://hdl.handle.net/11449/325069.
