# Supplementary material for: Effects of Extrusion Conditions and Oil Addition on the Characteristics of Cheese‐Flavored Corn Snacks and Food Bolus Formation and Properties
Source: J Texture Stud. 2026 Jul 5;57(4):e70102. doi: 10.1111/jtxs.70102 (PMC13333249; doi:10.1111/jtxs.70102)
Supplement: Supplementary file 1 — Table S1: Stimulated salivary flux and particle size of the carrot bolus (mean ± SD; n = 3). [file JTXS-57-e70102-s002.docx]

Supplementary Table 1: Stimulated salivary flux and particle size of the carrot bolus (mean ± SD; n = 3).

| Panelist | Salivary flux (mL/min) | Particle size (*d50*) (mm) |
| --- | --- | --- |
| P1 | 3.2 ± 0.4 | 11.6 ± 4.7 |
| P2 | 0.7 ± 0.1 | 12.2 ± 4.0 |
| P3 | 1.0 ± 0.1 | 9.9 ± 1.7 |
| P4 | 0.9 ± 0.1 | 6.4 ± 2.7 |
| P5 | 1.4 ± 0.0 | 11.2 ± 3.7 |
| P6 | 1.9 ± 0.1 | 12.8 ± 3.1 |
| P7 | 1.2 ± 0.0 | 9.8 ± 0.1 |
| P8 | 0.9 ± 0.3 | 7.8 ± 1.0 |
