# Supplementary material for: Effects of Extrusion Conditions and Oil Addition on the Characteristics of Cheese‐Flavored Corn Snacks and Food Bolus Formation and Properties
Source: J Texture Stud. 2026 Jul 5;57(4):e70102. doi: 10.1111/jtxs.70102 (PMC13333249; doi:10.1111/jtxs.70102)
Supplement: Supplementary file 2 — Table S2: Moisture (n = 3) and lipid (n = 2) content and physical parameters of corn snacks (n = 10). [file JTXS-57-e70102-s004.docx]

Supplementary Table 2: Moisture (n = 3) and lipid (n = 2) content and physical parameters of corn snacks (n = 10).

| Assay | M (%) | T (°C) | SO (%)* | Moisture (%) | Lipid (%) | Expansion ratio | Density (g/cm³) | Force peak (N) | Sound pressure level (dB) |
| --- | --- | --- | --- | --- | --- | --- | --- | --- | --- |
| 1 | 12 | 102 | 5 | 8.3 ± 0.3 | 4.7 ± 0.1 | 4.0 ± 0.2 | 0.14 ± 0.01 | 17.7 ± 2.6 | 90.7 ± 1.6 |
| 2 | 18 | 102 | 5 | 9.2 ± 0.1 | 5.5 ± 0.5 | 3.9 ± 0.2 | 0.14 ± 0.02 | 15.7 ± 2.4 | 89.9 ± 3.9 |
| 3 | 12 | 138 | 5 | 7.9 ± 0.1 | 4.6 ± 0.4 | 3.5 ± 0.2 | 0.11 ± 0.03 | 4.2 ± 1.7 | 73.1 ± 5.7 |
| 4 | 18 | 138 | 5 | 8.7 ± 0.5 | 3.4 ± 0.3 | 3.6 ± 0.2 | 0.10 ±0.01 | 6.4 ± 2.2 | 85.3 ± 3.5 |
| 5 | 12 | 102 | 19 | 8.0 ± 0.1 | 13.0 ± 0.1 | 4.1 ± 0.2 | 0.15 ±0.02 | 20.3 ± 5.8 | 94.2 ± 2.6 |
| 6 | 18 | 102 | 19 | 7.6 ± 0.2 | 12.6 ± 0.1 | 4.1 ± 0.1 | 0.13 ± 0.02 | 17.2 ± 5.3 | 95.0 ± 1.5 |
| 7 | 12 | 138 | 19 | 5.8 ± 0.0 | 12.9 ± 0.4 | 3.9 ± 0.4 | 0.13 ± 0.02 | 13.1 ± 5.9 | 82.5 ± 4.4 |
| 8 | 18 | 138 | 19 | 7.0 ± 0.3 | 15.0 ± 1.2 | 3.8 ± 0.4 | 0.10 ± 0.02 | 8.5 ± 3.5 | 81.9 ± 5.5 |
| 9 | 10 | 120 | 12 | 7.0 ± 0.3 | 8.3 ± 0.0 | 4.3 ± 0.3 | 0.11 ± 0.02 | 5.4 ± 2.5 | 79.5 ± 5.3 |
| 10 | 20 | 120 | 12 | 7.7 ± 0.1 | 7.8 ± 0.6 | 4.2 ± 0.2 | 0.12 ± 0.02 | 6.0 ± 6.5 | 69.6 ± 19.4 |
| 11 | 15 | 90 | 12 | 7.8 ± 0.1 | 12.2 ± 0.7 | 3.6 ± 0.3 | 0.18 ± 0.03 | 22.7 ± 7.9 | 84.1 ± 6.1 |
| 12 | 15 | 150 | 12 | 6.5 ± 0.1 | 10.3 ± 1.1 | 3.8 ± 0.3 | 0.11 ± 0.01 | 3.9 ± 3.2 | 61.3 ± 15.8 |
| 13 | 15 | 120 | 0 | 8.5 ± 0.2 | 0.7 ± 0.1 | 3.5 ± 0.2 | 0.11 ± 0.02 | 11.2 ± 3.4 | 75.5 ± 4.5 |
| 14 | 15 | 120 | 24 | 6.6 ± 0.3 | 16.7 ± 0.3 | 4.7 ± 0.2 | 0.10 ± 0.01 | 14.3 ± 4.4 | 91.7 ± 3.1 |
| 15 | 15 | 120 | 12 | 7.6 ± 0.2 | 8.8 ± 0.3 | 4.3 ± 0.2 | 0.13 ± 0.02 | 13.4 ± 4.0 | 87.2 ± 2.6 |
| 16 | 15 | 120 | 12 | 8.0 ± 0.1 | 11.4 ± 0.8 | 4.6 ± 0.2 | 0.11 ± 0.01 | 12.2 ± 3.4 | 90.6 ± 5.0 |
| 17 | 15 | 120 | 12 | 8.0 ± 0.2 | 8.3 ± 0.1 | 4.1 ± 0.3 | 0.12 ± 0.03 | 13.4 ± 4.2 | 89.1 ± 4.2 |

M = Moisture of corn grits.

T = Temperature of zone 5 of the barrel.

SO = Sunflower oil.

*% (w/w) referring to 100 g of extrudate.

Mean ± standard deviation.
