# Supplementary material for: Effects of Extrusion Conditions and Oil Addition on the Characteristics of Cheese‐Flavored Corn Snacks and Food Bolus Formation and Properties
Source: J Texture Stud. 2026 Jul 5;57(4):e70102. doi: 10.1111/jtxs.70102 (PMC13333249; doi:10.1111/jtxs.70102)
Supplement: Supplementary file 3 — Table S3: Properties of food boluses (mean ± standard deviation; n = 24) of corn snacks. [file JTXS-57-e70102-s003.docx]

**Supplementary Table 3:** Properties of food boluses (mean ± standard deviation; n = 24) of corn snacks.

| Assay | M (%) | T (°C) | SO (%)* | Chewing time (s) | Chewing cycles | Moisture of the food bolus (%) | Force peak (N) |
| --- | --- | --- | --- | --- | --- | --- | --- |
| 1 | 12 | 102 | 5 | 14.3 ± 3.8 | 18.5 ± 3.3 | 30.2 ± 10.5 | 0.20 ± 0.12 |
| 2 | 18 | 102 | 5 | 14.6 ± 3.7 | 19.4 ± 4.5 | 28.9 ± 9.6 | 0.24 ± 0.23 |
| 3 | 12 | 138 | 5 | 14.2 ± 2.8 | 15.8 ± 3.7 | 28.6 ± 10.2 | 0.26 ± 0.23 |
| 4 | 18 | 138 | 5 | 14.0 ± 3.5 | 17.5 ± 3.5 | 26.8 ± 10.0 | 0.24 ± 0.17 |
| 5 | 12 | 102 | 19 | 13.8 ± 3.4 | 19.5 ± 4.3 | 29.0 ± 10.4 | 0.19 ± 0.17 |
| 6 | 18 | 102 | 19 | 14.3 ± 3.4 | 19.3 ± 4.2 | 28.2 ± 10.2 | 0.17 ± 0.09 |
| 7 | 12 | 138 | 19 | 14.0 ± 3.4 | 16.3 ± 2.9 | 25.3 ± 7.8 | 0.22 ± 0.17 |
| 8 | 18 | 138 | 19 | 13.8 ± 3.8 | 16.8 ± 3.6 | 27.4 ± 10.6 | 0.19 ± 0.12 |
| 9 | 10 | 120 | 12 | 15.0 ± 3.0 | 17.4 ± 3.9 | 26.5 ± 9.7 | 0.27 ± 0.25 |
| 10 | 20 | 120 | 12 | 14.9 ± 3.0 | 17.1 ± 3.6 | 27.9 ± 10.4 | 0.29 ± 0.22 |
| 11 | 15 | 90 | 12 | 17.3 ± 4.0 | 19.1 ± 3.6 | 31.3 ± 12.2 | 0.21 ± 0.19 |
| 12 | 15 | 150 | 12 | 13.8 ± 3.1 | 15.8 ± 3.9 | 25.2 ± 8.2 | 0.30 ± 0.25 |
| 13 | 15 | 120 | 0 | 14.5 ± 2.9 | 16.7 ± 3.7 | 26.6 ± 9.1 | 0.32 ± 0.24 |
| 14 | 15 | 120 | 24 | 15.0 ± 4.7 | 17.8 ± 3.3 | 28.4 ± 13.4 | 0.21 ± 0.13 |
| 15 | 15 | 120 | 12 | 15.7 ± 3.2 | 16.5 ± 2.8 | 27.2 ± 11.1 | 0.19 ± 0.11 |
| 16 | 15 | 120 | 12 | 15.5 ± 3.9 | 17.7 ± 3.0 | 24.8 ± 9.3 | 0.17 ± 0.09 |
| 17 | 15 | 120 | 12 | 15.3 ± 3.4 | 17.2 ± 2.8 | 28.3 ± 9.9 | 0.23 ± 0.20 |

M = Moisture of corn grits.

T = Temperature of zone 5 of the barrel.

SO = Sunflower oil.

*% (w/w) referring to 100 g of extrudate.
