# Supplementary material for: Effects of Extrusion Conditions and Oil Addition on the Characteristics of Cheese‐Flavored Corn Snacks and Food Bolus Formation and Properties
Source: J Texture Stud. 2026 Jul 5;57(4):e70102. doi: 10.1111/jtxs.70102 (PMC13333249; doi:10.1111/jtxs.70102)
Supplement: Supplementary file 4 — Table S4: Particles of food boluses (mean ± standard deviation; n = 24) of corn snacks. [file JTXS-57-e70102-s001.docx]

**Supplementary Table 4:** Particles of food boluses (mean ± standard deviation; n = 24) of corn snacks.

| Assay | M (%) | T (°C) | OS (%)* | Particle number | Area (mm²) | Perimeter (mm) | Min Feret diameter (mm) | Max Feret diameter (mm) | Circularity |
| --- | --- | --- | --- | --- | --- | --- | --- | --- | --- |
| 1 | 12 | 102 | 5 | 96.0 ± 160.8 | 0.5 ± 0.1 | 11.6 ± 2.5 | 0.9 ± 0.4 | 1.7 ± 0.2 | 0.05 ± 0.02 |
| 2 | 18 | 102 | 5 | 101.5 ± 166.2 | 1.0 ± 2.6 | 14.7 ± 12.3 | 1.1 ± 0.4 | 1.7 ± 0.7 | 0.05 ± 0.02 |
| 3 | 12 | 138 | 5 | 197.4 ± 256.2 | 0.7 ± 0.6 | 14.4 ± 6.5 | 1.0 ± 0.6 | 1.8 ± 0.3 | 0.05 ± 0.01 |
| 4 | 18 | 138 | 5 | 153.3 ± 277.0 | 1.0 ±2.5 | 14.3 ± 6.9 | 1.1 ± 0.8 | 1.8 ± 0.6 | 0.05 ± 0.01 |
| 5 | 12 | 102 | 19 | 171.4 ± 332.6 | 6.2 ± 27.4 | 17.7 ± 23.6 | 1.3 ± 2.3 | 2.2 ± 1.6 | 0.05 ± 0.02 |
| 6 | 18 | 102 | 19 | 100.7 ± 121.6 | 0.5 ± 0.3 | 11.2 ±3.7 | 0.9 ± 0.3 | 1.6 ± 0.3 | 0.05 ± 0.02 |
| 7 | 12 | 138 | 19 | 167.3 ± 284.4 | 0.9 ± 1.7 | 12.8 ± 2.8 | 1.0 ± 0.3 | 1.7 ± 0.2 | 0.05 ± 0.01 |
| 8 | 18 | 138 | 19 | 78.8 ± 102.3 | 8.3 ± 22.5 | 27.4 ± 34.2 | 2.0 ± 3.6 | 3.2 ± 2.5 | 0.05 ± 0.02 |
| 9 | 10 | 120 | 12 | 202.0 ± 293.6 | 16.1 ± 73.1 | 30.9 ± 75.4 | 1.8 ± 6.5 | 3.3 ± 3.3 | 0.05 ± 0.03 |
| 10 | 20 | 120 | 12 | 94.5 ± 159.9 | 1.0 ± 94.5 | 13.9 ± 6.4 | 1.1 ± 0.7 | 1.8 ± 0.4 | 0.06 ± 0.02 |
| 11 | 15 | 90 | 12 | 107.6 ± 178.0 | 22.9 ± 1.0 | 31.9 ± 63.8 | 2.2 ± 6.0 | 3.6 ± 4.0 | 0.06 ± 0.02 |
| 12 | 15 | 150 | 12 | 113.2 ± 169.4 | 8.1 ± 74.1 | 20.3 ± 27.3 | 1.6 ± 3.3 | 2.7 ± 2.3 | 0.05 ± 0.02 |
| 13 | 15 | 120 | 0 | 72.0 ± 189.6 | 22.8 ± 66.5 | 36.1 ± 56.1 | 2.6 ± 5.1 | 3.8 ± 4.2 | 0.05 ± 0.01 |
| 14 | 15 | 120 | 24 | 166.5 ± 215.9 | 1.3 ± 3.1 | 14.6 ± 10.2 | 1.2 ± 1.0 | 1.9 ± 0.8 | 0.05 ± 0.02 |
| 15 | 15 | 120 | 12 | 107.4 ± 223.7 | 0.5 ± 0.3 | 12.2 ± 4.2 | 0.9 ± 0.6 | 1.8 ± 0.3 | 0.05 ± 0.02 |
| 16 | 15 | 120 | 12 | 144.7 ± 245.7 | 0.5 ± 0.2 | 11.7 ± 2.9 | 0.9 ± 0.3 | 1.7 ± 0.2 | 0.06 ± 0.04 |
| 17 | 15 | 120 | 12 | 93.6 ± 110.5 | 9.4 ± 42.9 | 18.8 ± 33.2 | 1.6 ± 3.9 | 2.4 ± 2.9 | 0.06 ± 0.02 |

M = Moisture of corn grits.

T = Temperature of zone 5 of the barrel.

SO = Sunflower oil.

*% (w/w) referring to 100 g of extrudate.
